# NLRP3 Inflammasome and MS/EAE

**DOI:** 10.1155/2013/859145

**Published:** 2013-01-08

**Authors:** Makoto Inoue, Mari L. Shinohara

**Affiliations:** ^1^Department of Immunology, Duke University Medical Center, DUMC-3010, Durham, NC 27710, USA; ^2^Department of Molecular Genetics and Microbiology, Duke University Medical Center, DUMC-3010, Durham, NC 27710, USA

## Abstract

Inflammasomes are cytosolic sensors that detect pathogens and danger signals in the innate immune system. The NLRP3 inflammasome is currently the most fully characterized inflammasome and is known to detect a wide array of microbes and endogenous damage-associated molecules. Possible involvement of the NLRP3 inflammasome (or inflammasomes) in the development of multiple sclerosis (MS) was suggested in a number of studies. Recent studies showed that the NLRP3 inflammasome exacerbates experimental autoimmune encephalomyelitis (EAE), an animal model of MS, although EAE can also develop without the NLRP3 inflammasome. In this paper, we discuss the NLRP3 inflammasome in MS and EAE development.

## 1. Inflammasomes

Inflammasomes are cytosolic sensors that detect pathogens and stresses in order to mature and secrete proinflammatory cytokines, such as interleukin-1*β* (IL-1*β*) and IL-18. Inflammasomes are expressed in phagocytes, such as macrophages and dendritic cells (DCs), and form a multiprotein complex that activates caspase-1. Assembly of inflammasomes that have clear physiological functions *in vivo* has been reported with relatively few NOD-like receptor (NLR) family members, such as NLRP1, NLRP3 (also called cryopyrin, CIAS1, NALP3), NLRC4 (IRAF), and AIM2 [1]. 

**Figure 1:**
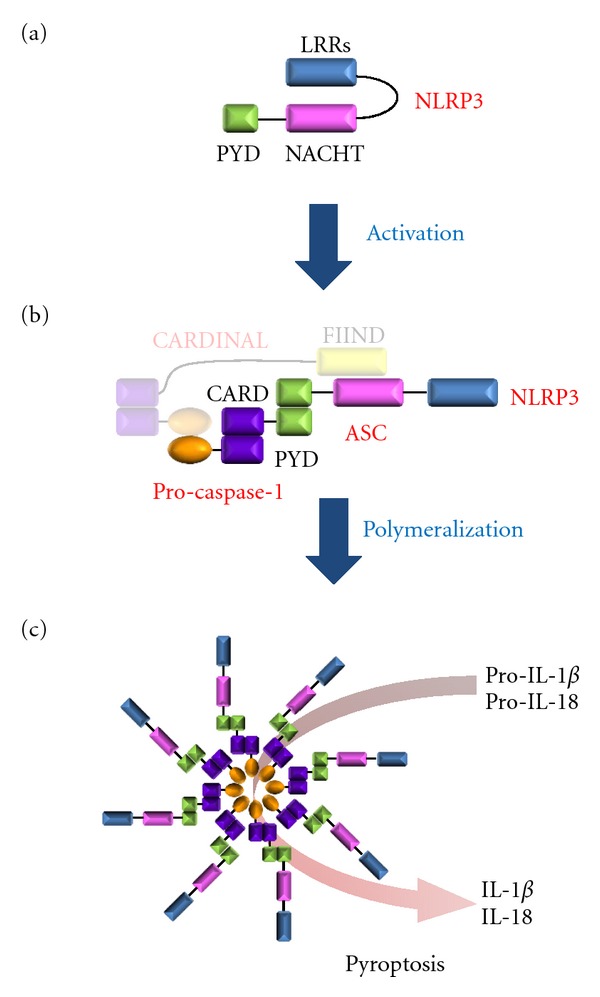
Assembly of the NLRP3 inflammasome. (a) Without an activator, NLRP3 is autorepressed by the interaction between LRRs and the NACHT domain. (b) NLRP3 activator opens up NLRP3 and allows to interact with ASC. ASC further interacts with procaspase-1. Although a CARDINAL homolog is not present in mice, we showed human CARDINAL, shown with a shade, as a part of the human NLRP3 inflammasome complex. (c) NLRP3 units polymerize. Active caspase-1 processes maturation of IL-1*β* and IL-18. Pyroptosis is also induced by activated caspase-1.

The NLRP3 inflammasome is currently the most fully characterized inflammasome and is comprised of three different proteins: NLRP3, adapter protein apoptosis-associated speck-like protein (ASC), and procaspase-1. NLRP3 protein is autorepressed by an internal interaction between the NACHT domain and leucine-rich repeats (LRRs) (Figure 1(a)) [2, 3]. Derepression of NLRP3 is essential for the interaction between NLRP3 and ASC through their Pyrin domains (PYD), followed by further interaction between ASC and procaspase-1 through CARD domains (caspase activation and recruitment domains) (Figure 1(b)). Oligomerization of the NLRP3 inflammasome heterotrimer unit leads to procaspase-1 self-cleavage to generate activated caspase-1, which processes maturation of IL-1*β* and IL-18 and elicits rapid release of those inflammatory cytokines by cell death termed “pyroptosis” (Figure 1(c)). Molecular mechanism by which caspase-1 mediates pyroptosis is still elusive, but is distinguished from apoptosis and necrosis [4, 5]. A molecule termed CARDINAL is known to be involved in the human NLRP3 inflammasome [6] (Figure 1(b)); but its function is unknown and there is no mouse homolog of human CARDINAL. Critical role of CARDINAL in eliciting functions of inflammasomes is questioned, because mouse inflammasomes share basic biological functions with human inflammasomes, despite the absence of CARDINAL. It is, at least, known that CARDINAL is dispensable for IL-1*β* production in human cells [7].

NLRP3 inflammasome senses various pathogens and damage-associated molecules, including viruses, bacteria, fungi, extracellular ATP, amyloid *β*, and uric acid [8–11], and various environmental irritants, such as silica, asbestos, and alum, also activate the NLRP3 inflammasome [3, 12, 13]. Studies showed that those stimuli generally induce reactive oxygen species (ROS) generation, the leakage of cathepsins from damaged lysosomes, or the efflux of potassium from cells by the loss of cell membrane integrity; and such intracellular molecules activate the NLRP3 inflammasome [8, 13–19]. However, the molecular mechanisms by which the NLRP3 inflammasome is activated by cathepsins, ROS, and the potassium efflux are also still not clear.

## 2. NLRP3 Inflammasome and MS

The involvement of NLRP3 inflammasome in human diseases was identified by studies showing constitutively active forms of NLRP3 by mutations within the *Nlrp3 *locus. The mutations correlate to autoinflammatory syndromes, such as Muckle-Wells syndrome (MWS), familial cold autoinflammatory syndrome, and cryopyrin-associated periodic syndrome [20–22]. Recent studies further revealed the involvement of the NLRP3 inflammasome in various human diseases, such as gout, type-2 diabetes, atherosclerosis, and inflammatory bowel diseases. Details of those are described in recent excellent reviews [23–26].

Multiple sclerosis (MS) is an autoimmune inflammatory demyelinating disease of the central nervous systems (CNSs) mediated by myelin-specific autoreactive T cells. There are a number of reports that strongly suggest the involvement of the NLRP3 inflammasome (or inflammasomes) in the development of MS. MS-like lesions were observed in a MWS patient who had a disease-susceptible mutation in the *Nlrp3 *gene [27, 28]. Expression of caspase-1 is elevated in MS plaques and peripheral blood mononuclear cells of MS patients [29, 30]. Abundance of caspase-1, together with that of IL-18, is also identified in peripheral mononuclear cells from MS patients compared to those cells from healthy controls [31]. It is of note that various human SNPs of caspase-1 have already been identified [32].

Quite a few studies showed correlation between severity of MS and IL-1*β* (and its receptor), which is a major cytokine processed by inflammasomes [33–40]. Levels of IL-1*β* in the cerebrospinal fluid (CSF) in MS patients are upregulated and correlated with susceptibility and progression of relapse-onset MS [33, 38, 39]. Treatment of MS patients with glatiramer acetate or IFN*β* is known to elevate the levels of endogenous IL-1 receptor antagonist [41, 42]. On the other hand, an NLRP3 inflammasome activator, ATP, is detected by a purinergic receptor, P2X7R. Elevated P2X7R expression in spinal cords of MS patients was observed [43, 44], and glatiramer acetate reduced the P2X7R expression levels [45]. Gain-of-function single nucleotide polymorphisms in the P2X7 receptor gene are considered to be associated to MS [46]. Those studies suggest the contribution of extracellular ATP to the development of MS. In addition to the levels of ATP, those of uric acid, which also activate the NLRP3 inflammasome, are also upregulated in the CSF of MS patients [47], and serum uric acid level in patients is potentially associated with susceptibility of MS [48]. It should be noted that increased cathepsin B activity in brains of MS patients was reported as well [49]. Because the word “inflammasomes” had not been used in earlier publications, the connection between MS and NLRP3 inflammasome was less visible. As we recently demonstrated the presence of an NLRP3 inflammasome-independent subset in experimental autoimmune encephalomyelitis (EAE), an animal model of MS [50], the NLRP3 inflammasome may not be involved in the development of all kinds of MS, which is a multifactorial and heterogeneous disease. However, these studies strongly suggest the general involvement of the NLRP3 inflammasome in MS. 

## 3. NLRP3 Inflammasome and EAE

**Figure 2:**
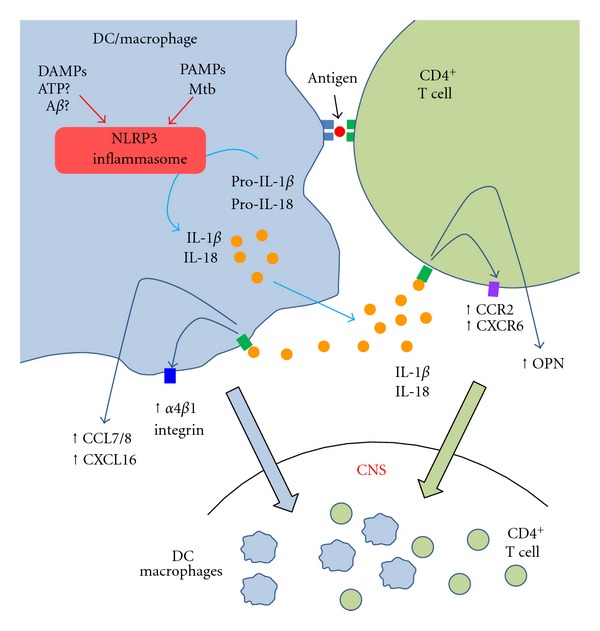
Role of NLRP3 inflammasome in the development of the autoimmune responses in EAE. Upon activation by DAMPs and possibly by PAMPs, the NLRP3 inflammasome processes IL-1*β* and IL-18 maturation in macrophages and DCs, which work as APCs. Secreted IL-1*β* and IL-18 were detected both by APCs as an autocrine manner and by CD4^+^ T cells, which are being activated with a self-antigen by the APCs. IL-1*β* and IL-18 induce expression of chemokines and their receptors both by T cells and APCs. Upregulation of chemokines and their receptors enhances migration of T cells and APCs to the CNS to progress EAE.

Ting and colleagues first reported the critical role of the NLRP3 inflammasome in EAE development using mice lacking gene encoding NLRP3 (*Nlrp3* ^−/−^) [51]. *Nlrp3* ^−/−^ mice displayed significantly mild EAE and reduction in IFN*γ*- and IL-17-expressing T helper (Th1 and Th17, resp.) cells in the peripheral lymphoid tissues and the spinal cord [51, 52]. NLRP3 inflammasome induces demyelination both in the EAE model and a chemically induced demyelinating disease model [52, 53]. More recently, we demonstrated that the NLRP3 inflammasome induces EAE progression by enhancing chemotactic migration of T helper cells and antigen-presenting cells (APCs) into the CNS [52] (Figure 2). We also showed that *Asc* ^−/−^ mice, which lack the gene encoding ASC, showed similar phenotypes to those in *Nlrp3* ^−/−^ mice [50, 52]. Because ASC is shared with other inflammasomes than NLRP3, the results suggest the major contribution of the NLRP3 inflammasomes in EAE pathogenicity among inflammasomes. Caspase-1-deficient mice are also resistant to EAE, supporting the involvement of inflammasomes (most probably NLRP3 inflammasome) in EAE pathogenicity [54, 55].

A tie between the NLRP3 inflammasome and EAE development is also supported by a number of other studies demonstrating enhanced levels of caspase-1, IL-1*β*, and IL-18 during EAE development [50, 51, 54]. NLRP3 inflammasome requires activation to exert its function; therefore, we evaluated and confirmed the activation of the NLRP3 inflammasome during EAE progression by detecting active caspase-1 (p20) in splenocytes and in CNS and high levels of IL-1*β* and IL-18 in serum and in spleen [50]. IL-1R-deficient mice and mice treated with a recombinant IL-1R antagonist (IL-1Ra) displayed mild EAE with reduced numbers of Th17 cells [56, 57]. In addition, IL-18 expression is increased in serum from mice that develop EAE [51], and IL-18 expression in EAE mice depends on the presence of ASC and NLRP3 [50]. IL-18-deficient (*Il18* ^−/−^) mice display significantly mild EAE [51, 58]. Another report to suggest the involvement of the NLRP3 inflammasome in EAE is amelioration of the disease by P2X7 receptor blockade, which prevents cells from detecting ATP [59]. Taken together, these studies demonstrated that the NLRP3 inflammasome plays an important role in EAE.

## 4. Impact of IL-1*β* and IL-18 on EAE

IL-1*β* and IL-18 are cytokines matured by the NLRP3 inflammasome. Involvement of IL-1*β* and IL-18 in EAE progression had long been speculated. IL-1*β* plays a role in reversing demyelination and breakdown of blood-brain barrier, which prevents infiltration of T cells and other cells into the CNS in healthy individuals [60]. IL-1*β* also induces activation of microglia [61], which stimulate CNS-infiltrated T cells by presenting self-antigens during EAE development. IL-1*β*, together with IL-23, promotes IL-17 expression both by *γδ*T cells and CD4^+^ T cells [56, 62]. (In particular, the critical role of Th17 cells in EAE has been well documented.)

IL-18 has been studied in the context of EAE. High *Il18* mRNA levels were found in the brain and the spinal cord at the onset and throughout the course of EAE [63, 64]. There are conflicting results on EAE in IL-18-deficient mice. Two groups showed significantly mild EAE in *Il18* ^−/−^ mice by defects in mounting autoreactive Th1/Th17 and autoantibody responses [51, 58], but another showed only slightly milder EAE in *Il18* ^−/−^ mice compared to WT mice [65]. (The latter study also showed that IL-18 receptor-deficient mice were completely resistant to EAE.) In both studies, *Il18* ^−/−^ mice were reasonably backcrossed enough, and the backcross generations do not look to be a major issue. Rather, we suspect that the discrepancy could come from the distinct EAE inducition regimens among those studies. In a report that showed resistance to EAE in *Il18* ^−/−^ mice, transfer of splenocytes from immunized *Il18* ^−/−^ mice to recipients did not induce EAE [58]. The result is congruent with our finding that transfer of peripheral T cells from immunized *Nlrp3* ^−/−^ and *Asc* ^−/−^ mice to recipients failed to induce EAE [52]. These results suggest a pathogenic role of IL-18, which is matured by inflammasomes in APCs during the activation of peripheral T cells after immunization. NLRP3 inflammasome-mediated IL-18 production is also known to exacerbate demyelination [53] and to promote IL-17 production by *γδ*T cells as well as CD4^+^ T cells [66] during EAE progression. Based on these findings and the proinflammatory character of IL-1*β* and IL-18, both cytokines are involved in EAE progression.

## 5. EAE Development by Immune Cell Chemotaxis Induced by the NLRP3 Inflammasome

More recently, the NLRP3 inflammasome was shown to mediate EAE progression by inducing chemotactic ability of immune cells, rather than augmenting the Th17 cell population [52]. Reduced Th17 cell population in *Asc* ^−/−^ and *Nlrp3* ^−/−^ miceappears to be one of mechanisms by which the absence of the NLRP3 inflammasomes ameliorates EAE, but it is not a causal factor of the resistance to EAE in *Asc* ^−/−^ and *Nlrp3* ^−/−^ mice. Instead, *Asc* ^−/−^ and *Nlrp3* ^−/−^ mice developed mild EAE because Th17 cells generated in those mice were not equipped to migrate into the CNS [52]; therefore, the quality of Th17 cells matters in the resistance of *Asc* ^−/−^ and *Nlrp3* ^−/−^ mice to EAE, rather than the quantity of Th17 cells. Th17 cells (and other T helper cells) from *Asc* ^−/−^ and *Nlrp3* ^−/−^ mice express low levels of CCR2, CXCR6, and osteopontin (OPN) [52] (Figure 2). Interestingly, chemotaxis of APCs in immunized *Asc* ^−/−^ and *Nlrp3* ^−/−^ mice was also impaired by diminished gene expression of CCL7/MCP3 (CCR2 ligand), CCL8/MCP2 (CCR2 ligand), CXCL16 (CXCR6 ligand), and *α*4*β*1 integrin (OPN receptor) [52] (Figure 2). Intriguingly, those molecules that are upregulated during EAE development by the presence of the NLRP3 inflammasome are matching chemokine/receptor pairs between T cells and APCs. *In vitro *analyses to evaluate cellular chemotaxis indeed demonstrated that T cells and DCs from immunized *Asc* ^−/−^ and *Nlrp3* ^−/−^ mice were severely compromised in migration ability [52]. 

Roles of these chemokines and their receptors in EAE development were reported. Previous studies showed that *Ccr2* ^−/−^ mice recruit reduced numbers of mononuclear cells in the CNS and show mild EAE [67, 68]. Actually, CCR2 expression in circulating CD4^+^ T cells is significantly elevated during MS relapse [69, 70]. On the other hand, roles of CCR2 ligands, CCL7/MCP3 and CCL8/MCP2, in EAE are less characterized, compared to other proteins detected in our study [52], but some MS studies suggest the involvement of MCP2 and MCP3 in the disease development. For example, both MCP2 and MCP3 are considered to be involved in the development of MS lesions in the CNS [71]. A genetic polymorphism was also reported in the promoter-enhancer region of the MCP3 gene of MS patients [72]. CXCR6 is required for neuroinflammation by immune cell infiltration in cortical injury sites [73]. *Cxcr6* ^−/−^ mice are not resistant to EAE, but antibodies against CXCL16 is known to reduce EAE severity [73, 74]. OPN is widely known to be an chemoattractant for immune cells [75]. In addition to high expression of OPN in MS lesions [76], OPN-deficient mice develop milder EAE than WT mice [76–80]. OPN also induces Th17 responses [78, 81] and sustains inflammatory T cells by inhibiting apoptosis [79] to exacerbate EAE [82]. The *α*4*β*1 integrin, an OPN receptor, in induction of EAE and MS has been well characterized. Indeed, an FDA-approved MS drug, natalizumab, is a humanized monoclonal antibody against *α*4 integrin [83, 84]. 

## 6. Two Subtypes of EAE: NLRP3 Dependent or Independent

Two initial reports on the NLRP3 inflammasome in EAE showed contrasting results. One showed susceptibility to EAE of *Nlrp3* ^−/−^ mice [85] and the other showed resistance of *Nlrp3* ^−/−^ mice [51]. The results appeared to be conflicting, but they do not: EAE can be induced with or without the NLRP3 inflammasome depending on the intensity of immunization [50]. Such NLRP3 inflammasome-independent EAE can be induced by aggressive immunization. For example, high doses of heat-killed *Mycobacteria *in complete Freund's adjuvant (CFA) are sufficient to induce EAE in *Nlrp3* ^−/−^ and *Asc* ^−/−^ mice [50]. Similar observation was reported in a study of caspase-1-deficient mice, in which disease susceptibility is associated with the number of immunization, the dose, and the MHC-binding affinity of antigen peptide [54]. Therefore, intensive antigen presentation by APCs to T cells appears to break tolerance and this might induce NLRP3 inflammasome-independent EAE. On the other hand, passive EAE, induced by an adoptive transfer of activated T cells to recipient mice, is NLRP3 inflammasome-dependent [50]. As passive EAE induction does not include CFA, the result suggests that adjuvant is not essential for activating the NLRP3 inflammasome. Importantly, we further demonstrated that the treatment of IFN*β*, which is a first-line treatment for MS, is not effective when EAE progression is independent of the NLRP3 inflammasome [50]. In human MS disease, such an artificial disease induction is not involved; therefore, it is currently not clear whether and how MS progresses in an NLRP3 inflammasome-independent fashion.

## 7. Conclusion and Perspectives

Although EAE is an excellent model to understand MS, EAE and MS are not the same disease and the findings in EAE may not be able to fully apply to MS. However, without using animal models, it is almost impossible to understand the role of inflammasomes in the development of those neuroinflammatory diseases. Congruent with a number of studies on MS patients, animal EAE models strongly suggested the involvement of inflammasomes, NLRP3 in particular, in autoimmune neuroinflammation. Because of the relatively recent identification of the structure of inflammasomes and its characterization, many studies of MS and EAE did not mention “inflammasomes,” despite descriptions of caspase-1, IL-1*β*, and other inflammasome-related molecules. As discussed in this paper, now we started to understand the role of the NLRP3 inflammasome in EAE development. To develop better MS treatments, it may be necessary to understand innate inflammation. Mechanistic understanding of innate inflammation caused by the NLRP3 inflammasome using EAE models and testing hypotheses obtained from EAE studies in MS are expected to bring a considerable progress in the efforts to treat MS.
